# [1,3-Bis(2,6-diiso­propyl­phen­yl)-1,3-di­hydro-2*H*-imidazol-2-yl­idene]tri­iodo­borane benzene hemisolvate

**DOI:** 10.1107/S2414314620008639

**Published:** 2020-06-30

**Authors:** Frauke Schödel, Hans-Wolfram Lerner, Michael Bolte

**Affiliations:** a Institut für Anorganische und Analytische Chemie, Goethe-Universität Frankfurt, Max-von-Laue-Strasse 7, 60438 Frankfurt am Main, Germany; University of Aberdeen, Scotland

**Keywords:** crystal structure, NHC, borane

## Abstract

In the title hemisolvate, C_27_H_36_BI_3_N_2_^.^0.5C_6_H_6_, the dihedral angles between the central heterocyclic ring and pendant benzene rings are 82.9 (8) and 88.7 (9)° and the complete benzene solvent mol­ecule of crystallization is generated by a crystallographic centre of inversion. In the crystal, one very weak C—H⋯I inter­action links the mol­ecules into [001] chains.

## Abstract 2

In the title hemisolvate, the dihedral angles between the central heterocyclic ring and pendant benzene rings are 82.9 (8) and 88.7 (9)° and the complete benzene solvent mol­ecule of crystallization is generated by a crystallographic centre of inversion. In the crystal, one very weak C—H⋯I inter­action links the mol­ecules into [001] chains.

## Graphical abstract

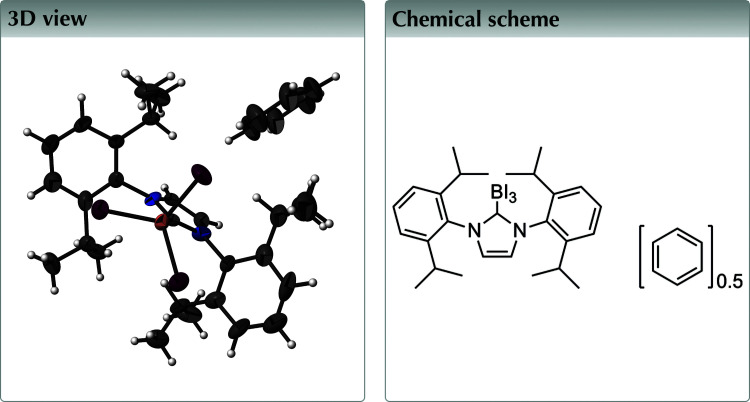

## Structure description

**Figure 1:**
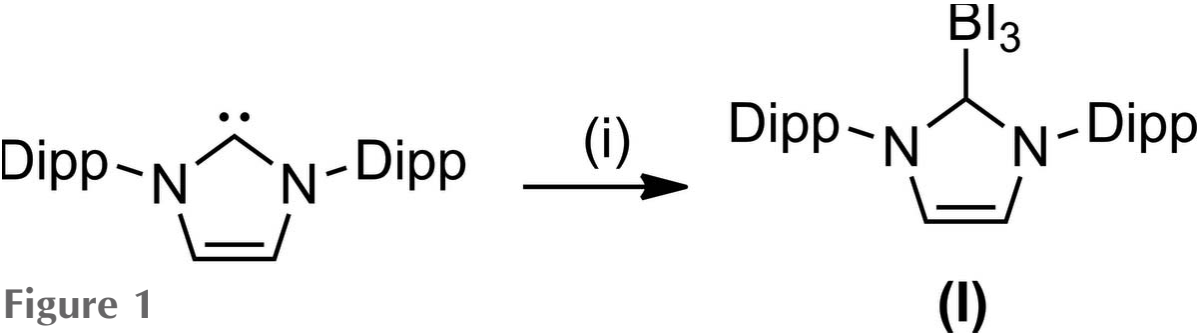
Reaction scheme for the preparation of the title compound.

NHC-complexed trihaloboranes B*X*_3_^.^NHC_IPr_ (NHC_IPr_ = IPr) are of great technological importance, because they find use in the synthesis of borsubhalides and also as precursor mol­ecules for B=B double-bond-containing diborenes (Wang *et al.*, 2007 ▸). In the course of our investigations of silanides (Lerner, 2005 ▸; Budanow *et al.*, 2014 ▸), we treated trihalides of the type *EX*_3_ (*E* = B, Al, Ga; *X* = Cl, Br) with the NHC supersilyl silver complex [Ag(IPr)Si*t*Bu_3_] (Schödel *et al.*, 2020 ▸). It is remarkable that along with *t*Bu_3_SiE*X*_2_, *EX*_3_·IPr was thereby formed. The identity of *EX*_3_·IPr was confirmed by comparison with authentic samples that were obtained by an equimolar reaction of *EX*_3_ with NHC_IPr_ (Schödel *et al.*, 2020 ▸). We now describe the synthesis and structure of the NHC-complexed tri­iodo­borane, BI_3_·IPr, which can be prepared by an analogous approach from NHC_IPr_ and BI_3_, as shown in Fig. 1 ▸.

**Figure 2:**
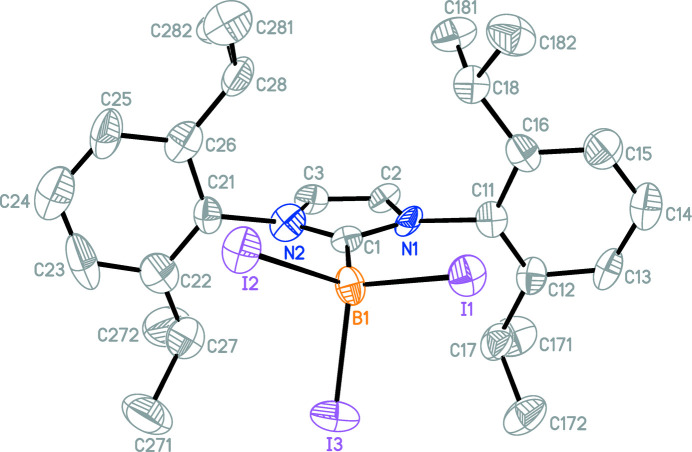
A perspective view of the NHC-mol­ecule in the title compound; H atoms and the solvent benzene mol­ecule have been omitted for clarity. Displacement ellipsoids are drawn at the 50% probability level.

The mol­ecular structure of the title compound (Fig. 2 ▸) does not show any unusual features: the C1—B1 bond length is 1.63 (2) Å and the dihedral angles between the central C1/C2/C3/N1/N2 heterocyclic ring and the pendant C11–C16 and C21–C26 benzene rings are 82.9 (8) and 88.7 (9)°, respectively. The complete benzene solvent mol­ecule is generated by a crystallographic centre of inversion. The structures of the tri­fluoro-substituted borane (Bolte *et al.*, 2020 ▸), which crystallizes without any solvent in the triclinic space group *P* 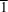 , and that of the tri­bromo-substituted borane (Wang *et al.*, 2007 ▸; Wang & Robinson, 2011 ▸), which crystallizes without any solvent in the monoclinic space group *P*2_1_/*n*, agree well with that of the title compound.

The only directional inter­action identified in the title compound is a very weak C—H⋯I inter­action (Table 1 ▸), which links the mol­ecules into *C*(6) [001] chains.

## Synthesis and crystallization

The NHC-complexed tri­iodo­borane (I) was synthesized according to a synthesis protocol for halogenated NHC-complexes (Schödel *et al.*, 2020 ▸). Treatment of a mixture of BI_3_ (85 mg, 0.22 mmol) in 6 ml benzene/hexane with NHC_IPr_ (IPr) (76 mg, 0.20 mmol) yielded qu­anti­tatively (I).

After pipetting from insoluble material, single crystals of (I) were grown from the reaction solution (benzene/hexa­ne) at room temperature.

## Refinement

Crystal data, data collection and structure refinement details are summarized in Table 2 ▸. Some significant peaks remain in the final electron difference map close to the I atoms.

## Supplementary Material

Crystal structure: contains datablock(s) I. DOI: 10.1107/S2414314620008639/hb4351sup1.cif

Structure factors: contains datablock(s) I. DOI: 10.1107/S2414314620008639/hb4351Isup2.hkl

CCDC reference: 2012458

Additional supporting information:  crystallographic information; 3D view; checkCIF report

## Figures and Tables

**Table 1:** Hydrogen-bond geometry (Å, °)

*D*—H⋯*A*	*D*—H	H⋯*A*	*D*⋯*A*	*D*—H⋯*A*
C2—H2⋯I2^i^	0.95	3.03	3.773 (13)	136

Symmetry code: (i) 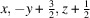 .

**Table 2:** Experimental details

Crystal data	
Chemical formula	C_27_H_36_BI_3_N_2_·0.5C_6_H_6_
*M*_r_	819.14
Crystal system, space group	Monoclinic, *P*2_1_/*c*
Temperature (K)	173
*a*, *b*, *c* (Å)	16.0100 (14), 12.5361 (11), 16.3086 (18)
β (°)	90.041 (8)
*V* (Å^3^)	3273.2 (5)
*Z*	4
Radiation type	Mo *K*α
μ (mm^−1^)	2.89
Crystal size (mm)	0.21 × 0.18 × 0.18

Data collection	
Diffractometer	STOE IPDS II two-circle diffractometer
Absorption correction	Multi-scan (*X-AREA*; Stoe & Cie, 2001 ▸)
*T*_min_, *T*_max_	0.719, 1.000
No. of measured, independent and observed [*I* > 2σ(*I*)] reflections	11917, 5990, 4253
*R*_int_	0.051
(sin θ/λ)_max_ (Å^−1^)	0.609

Refinement	
*R*[*F* ^2^ > 2σ(*F* ^2^)], *wR*(*F* ^2^), *S*	0.094, 0.267, 1.13
No. of reflections	5990
No. of parameters	325
H-atom treatment	H-atom parameters constrained
	
Δρ_max_, Δρ_min_ (e Å^−3^)	6.06, −1.70

Computer programs: *X-AREA* (Stoe & Cie, 2001 ▸), *XP* in *SHELXTL-Plus* and *SHELXS* (Sheldrick, 2008 ▸), *SHELXL2016/6* (Sheldrick, 2015 ▸) and *publCIF* (Westrip, 2010 ▸).

## full crystallographic data

### Crystal data

**Table:**

C_27_H_36_BI_3_N_2_·0.5C_6_H_6_	*F*(000) = 1588
*M**_r_* = 819.14	*D*_x_ = 1.662 Mg m^−^^3^
Monoclinic, *P*2_1_/*c*	Mo *K*α radiation, λ = 0.71073 Å
*a* = 16.0100 (14) Å	Cell parameters from 13397 reflections
*b* = 12.5361 (11) Å	θ = 3.5–25.9°
*c* = 16.3086 (18) Å	µ = 2.89 mm^−^^1^
β = 90.041 (8)°	*T* = 173 K
*V* = 3273.2 (5) Å^3^	Block, colourless
*Z* = 4	0.21 × 0.18 × 0.18 mm

### Data collection

**Table:**

STOE IPDS II two-circle-diffractometer	4253 reflections with *I* > 2σ(*I*)
Radiation source: Genix 3D IµS microfocus X-ray source	*R*_int_ = 0.051
ω scans	θ_max_ = 25.6°, θ_min_ = 3.5°
Absorption correction: multi-scan (X-Area; Stoe & Cie, 2001)	*h* = −19→19
*T*_min_ = 0.719, *T*_max_ = 1.000	*k* = −15→12
11917 measured reflections	*l* = −16→19
5990 independent reflections	

### Refinement

**Table:**

Refinement on *F*^2^	Primary atom site location: structure-invariant direct methods
Least-squares matrix: full	Hydrogen site location: inferred from neighbouring sites
*R*[*F*^2^ > 2σ(*F*^2^)] = 0.094	H-atom parameters constrained
*wR*(*F*^2^) = 0.267	*w* = 1/[σ^2^(*F*_o_^2^) + (0.1189*P*)^2^ + 68.6175*P*] where *P* = (*F*_o_^2^ + 2*F*_c_^2^)/3
*S* = 1.13	(Δ/σ)_max_ < 0.001
5990 reflections	Δρ_max_ = 6.06 e Å^−^^3^
325 parameters	Δρ_min_ = −1.70 e Å^−^^3^
0 restraints	

### Special details

**Table:**

Geometry. All esds (except the esd in the dihedral angle between two l.s. planes) are estimated using the full covariance matrix. The cell esds are taken into account individually in the estimation of esds in distances, angles and torsion angles; correlations between esds in cell parameters are only used when they are defined by crystal symmetry. An approximate (isotropic) treatment of cell esds is used for estimating esds involving l.s. planes.
Refinement. The H atoms were refined using a riding model (C—H = 0.95–1.00 Å) and with *U*_iso_(H) = 1.2*U*_eq_(C) or *U*_iso_(H) = 1.5*U*_eq_(C_methyl_).

### Fractional atomic coordinates and isotropic or equivalent isotropic displacement parameters (Å^2^)

**Table:**

	*x*	*y*	*z*	*U*_iso_*/*U*_eq_	
B1	0.2324 (12)	0.7430 (14)	0.2487 (11)	0.033 (4)	
I1	0.12197 (7)	0.67550 (9)	0.18663 (6)	0.0363 (3)	
I2	0.25388 (8)	0.90540 (9)	0.19351 (6)	0.0406 (3)	
I3	0.34318 (8)	0.63957 (11)	0.21405 (7)	0.0454 (4)	
N1	0.1868 (8)	0.6784 (9)	0.3986 (7)	0.028 (3)	
N2	0.2796 (9)	0.8040 (10)	0.3982 (7)	0.035 (3)	
C1	0.2319 (9)	0.7468 (11)	0.3483 (8)	0.025 (3)	
C2	0.2103 (10)	0.6921 (11)	0.4788 (8)	0.027 (3)	
H2	0.189436	0.654241	0.524932	0.032*	
C3	0.2693 (10)	0.7701 (13)	0.4796 (8)	0.031 (3)	
H3	0.298079	0.796690	0.526316	0.038*	
C11	0.1170 (10)	0.6030 (12)	0.3801 (8)	0.029 (3)	
C12	0.1357 (10)	0.4968 (12)	0.3790 (9)	0.034 (4)	
C13	0.0698 (12)	0.4282 (13)	0.3723 (10)	0.042 (4)	
H13	0.080143	0.353572	0.371122	0.051*	
C14	−0.0126 (13)	0.4652 (16)	0.3671 (12)	0.051 (5)	
H14	−0.057738	0.416627	0.361099	0.061*	
C15	−0.0266 (13)	0.5719 (16)	0.3709 (11)	0.049 (5)	
H15	−0.082456	0.597269	0.368245	0.059*	
C16	0.0370 (10)	0.6443 (15)	0.3785 (9)	0.038 (4)	
C17	0.2240 (12)	0.4532 (14)	0.3828 (12)	0.046 (4)	
H17	0.264021	0.514429	0.383612	0.055*	
C18	0.0197 (11)	0.7668 (13)	0.3826 (9)	0.036 (4)	
H18	0.069516	0.804400	0.359575	0.043*	
C21	0.3265 (10)	0.9006 (13)	0.3832 (9)	0.032 (3)	
C22	0.4127 (12)	0.8955 (16)	0.3719 (10)	0.047 (4)	
C23	0.4507 (14)	0.992 (2)	0.3564 (12)	0.066 (7)	
H23	0.508706	0.992851	0.343977	0.079*	
C24	0.4077 (18)	1.0863 (19)	0.3581 (14)	0.069 (6)	
H24	0.435766	1.151592	0.347351	0.082*	
C25	0.3235 (15)	1.0863 (16)	0.3755 (12)	0.055 (5)	
H25	0.294598	1.152424	0.376634	0.066*	
C26	0.2808 (13)	0.9963 (14)	0.3908 (9)	0.042 (4)	
C27	0.4606 (13)	0.7956 (17)	0.3801 (11)	0.052 (5)	
H27	0.421405	0.733606	0.376851	0.062*	
C28	0.1927 (13)	0.9982 (14)	0.4164 (11)	0.046 (4)	
H28	0.169521	0.924186	0.413065	0.055*	
C171	0.2336 (16)	0.3898 (18)	0.4635 (13)	0.064 (6)	
H17A	0.220421	0.436203	0.510040	0.095*	
H17B	0.195372	0.328798	0.463031	0.095*	
H17C	0.291254	0.364193	0.468496	0.095*	
C172	0.2441 (15)	0.3830 (14)	0.3101 (13)	0.056 (5)	
H17D	0.301550	0.356578	0.315018	0.084*	
H17E	0.205436	0.322448	0.308768	0.084*	
H17F	0.238512	0.424393	0.259455	0.084*	
C181	0.0110 (14)	0.7997 (16)	0.4721 (12)	0.053 (5)	
H18A	0.060730	0.777301	0.502604	0.080*	
H18B	0.005069	0.877349	0.475562	0.080*	
H18C	−0.038561	0.765534	0.495714	0.080*	
C182	−0.0563 (12)	0.7999 (18)	0.3326 (12)	0.054 (5)	
H18D	−0.048754	0.777605	0.275433	0.082*	
H18E	−0.106342	0.765724	0.355077	0.082*	
H18F	−0.062712	0.877539	0.334926	0.082*	
C271	0.5312 (15)	0.782 (2)	0.3144 (17)	0.080 (8)	
H27A	0.506499	0.781796	0.259435	0.120*	
H27B	0.571259	0.840351	0.319159	0.120*	
H27C	0.560099	0.713624	0.323616	0.120*	
C272	0.5046 (16)	0.794 (2)	0.4622 (17)	0.081 (8)	
H27D	0.536418	0.727802	0.467732	0.122*	
H27E	0.542640	0.855189	0.465828	0.122*	
H27F	0.463099	0.798586	0.506255	0.122*	
C281	0.1881 (16)	1.0382 (18)	0.5090 (11)	0.062 (6)	
H28A	0.129747	1.039843	0.526916	0.093*	
H28B	0.219884	0.989359	0.544107	0.093*	
H28C	0.212028	1.109989	0.512931	0.093*	
C282	0.1388 (14)	1.0719 (16)	0.3653 (11)	0.053 (5)	
H28D	0.081148	1.069398	0.385329	0.079*	
H28E	0.160093	1.144959	0.369588	0.079*	
H28F	0.140292	1.049016	0.307829	0.079*	
C31	0.4504 (16)	0.514 (3)	0.4308 (16)	0.082 (8)	
H31	0.416372	0.526785	0.384172	0.098*	
C32	0.4206 (16)	0.535 (2)	0.5071 (19)	0.084 (8)	
H32	0.364101	0.557238	0.512498	0.101*	
C33	0.4704 (15)	0.526 (2)	0.5791 (16)	0.075 (7)	
H33	0.449907	0.547160	0.631386	0.090*	

### Atomic displacement parameters (Å^2^)

**Table:**

	*U*^11^	*U*^22^	*U*^33^	*U*^12^	*U*^13^	*U*^23^
B1	0.032 (10)	0.033 (9)	0.032 (9)	−0.008 (7)	0.001 (7)	0.009 (7)
I1	0.0414 (6)	0.0459 (6)	0.0216 (5)	−0.0051 (5)	−0.0054 (4)	−0.0024 (4)
I2	0.0556 (8)	0.0415 (6)	0.0247 (5)	−0.0091 (5)	−0.0036 (4)	0.0046 (4)
I3	0.0407 (7)	0.0604 (8)	0.0352 (6)	0.0203 (6)	0.0017 (5)	−0.0040 (5)
N1	0.044 (8)	0.023 (6)	0.018 (6)	−0.007 (5)	0.003 (5)	−0.010 (5)
N2	0.054 (9)	0.032 (7)	0.020 (6)	0.001 (6)	−0.002 (6)	0.013 (5)
C1	0.030 (8)	0.024 (7)	0.022 (7)	0.005 (6)	0.000 (6)	0.000 (5)
C2	0.037 (9)	0.026 (7)	0.017 (6)	0.005 (6)	−0.011 (6)	−0.002 (5)
C3	0.037 (9)	0.041 (9)	0.015 (6)	0.003 (7)	−0.004 (6)	−0.006 (6)
C11	0.037 (9)	0.037 (8)	0.014 (6)	−0.004 (7)	−0.005 (6)	0.008 (6)
C12	0.040 (9)	0.027 (8)	0.034 (8)	−0.003 (7)	−0.007 (7)	0.011 (6)
C13	0.057 (12)	0.032 (9)	0.038 (9)	−0.013 (8)	−0.010 (8)	−0.005 (7)
C14	0.056 (13)	0.050 (11)	0.048 (11)	−0.019 (9)	−0.009 (9)	0.000 (9)
C15	0.051 (12)	0.058 (12)	0.038 (9)	−0.004 (9)	−0.003 (8)	−0.010 (8)
C16	0.033 (9)	0.058 (11)	0.023 (7)	−0.006 (8)	0.001 (6)	−0.003 (7)
C17	0.047 (11)	0.026 (8)	0.065 (12)	0.001 (8)	−0.007 (9)	0.000 (8)
C18	0.040 (10)	0.040 (9)	0.029 (8)	0.000 (7)	−0.005 (7)	0.005 (7)
C21	0.037 (9)	0.036 (8)	0.021 (7)	−0.011 (7)	0.000 (6)	0.003 (6)
C22	0.046 (11)	0.064 (12)	0.030 (8)	−0.008 (9)	−0.004 (8)	−0.007 (8)
C23	0.053 (13)	0.100 (19)	0.046 (11)	−0.045 (13)	0.004 (9)	0.000 (11)
C24	0.09 (2)	0.047 (12)	0.065 (14)	−0.018 (13)	−0.005 (13)	−0.001 (10)
C25	0.077 (16)	0.043 (11)	0.046 (11)	−0.027 (10)	0.000 (10)	−0.009 (8)
C26	0.066 (12)	0.038 (9)	0.021 (7)	−0.005 (9)	−0.002 (7)	0.000 (7)
C27	0.050 (11)	0.063 (13)	0.042 (10)	−0.009 (10)	−0.015 (8)	−0.014 (9)
C28	0.060 (13)	0.027 (8)	0.051 (10)	−0.010 (8)	0.006 (9)	−0.008 (8)
C171	0.076 (16)	0.063 (14)	0.051 (12)	0.021 (12)	−0.017 (11)	0.004 (10)
C172	0.084 (16)	0.030 (10)	0.055 (12)	0.004 (9)	0.003 (11)	−0.001 (8)
C181	0.062 (13)	0.044 (11)	0.054 (11)	0.016 (9)	−0.002 (10)	−0.004 (9)
C182	0.043 (11)	0.075 (14)	0.046 (10)	0.011 (10)	−0.009 (9)	−0.006 (10)
C271	0.047 (14)	0.11 (2)	0.086 (18)	0.002 (14)	0.019 (12)	−0.016 (16)
C272	0.065 (16)	0.087 (19)	0.093 (19)	0.011 (14)	−0.012 (14)	−0.030 (15)
C281	0.095 (18)	0.056 (13)	0.035 (10)	0.025 (12)	0.012 (10)	−0.002 (9)
C282	0.074 (14)	0.051 (11)	0.033 (9)	0.007 (10)	0.000 (9)	0.006 (8)
C31	0.059 (15)	0.12 (2)	0.063 (15)	0.023 (15)	−0.017 (12)	−0.002 (15)
C32	0.045 (13)	0.10 (2)	0.10 (2)	0.016 (13)	−0.012 (14)	0.015 (17)
C33	0.046 (13)	0.11 (2)	0.068 (15)	−0.002 (13)	0.001 (11)	−0.011 (14)

### Geometric parameters (Å, º)

**Table:**

B1—C1	1.63 (2)	C25—H25	0.9500
B1—I1	2.205 (18)	C26—C28	1.47 (3)
B1—I2	2.252 (17)	C27—C272	1.51 (3)
B1—I3	2.27 (2)	C27—C271	1.57 (3)
N1—C2	1.373 (17)	C27—H27	1.0000
N1—C1	1.389 (19)	C28—C282	1.51 (3)
N1—C11	1.494 (19)	C28—C281	1.59 (3)
N2—C1	1.326 (19)	C28—H28	1.0000
N2—C3	1.404 (18)	C171—H17A	0.9800
N2—C21	1.45 (2)	C171—H17B	0.9800
C2—C3	1.36 (2)	C171—H17C	0.9800
C2—H2	0.9500	C172—H17D	0.9800
C3—H3	0.9500	C172—H17E	0.9800
C11—C12	1.37 (2)	C172—H17F	0.9800
C11—C16	1.38 (2)	C181—H18A	0.9800
C12—C13	1.37 (2)	C181—H18B	0.9800
C12—C17	1.52 (2)	C181—H18C	0.9800
C13—C14	1.40 (3)	C182—H18D	0.9800
C13—H13	0.9500	C182—H18E	0.9800
C14—C15	1.36 (3)	C182—H18F	0.9800
C14—H14	0.9500	C271—H27A	0.9800
C15—C16	1.37 (3)	C271—H27B	0.9800
C15—H15	0.9500	C271—H27C	0.9800
C16—C18	1.56 (2)	C272—H27D	0.9800
C17—C172	1.51 (3)	C272—H27E	0.9800
C17—C171	1.54 (3)	C272—H27F	0.9800
C17—H17	1.0000	C281—H28A	0.9800
C18—C182	1.52 (2)	C281—H28B	0.9800
C18—C181	1.52 (2)	C281—H28C	0.9800
C18—H18	1.0000	C282—H28D	0.9800
C21—C22	1.39 (3)	C282—H28E	0.9800
C21—C26	1.41 (2)	C282—H28F	0.9800
C22—C23	1.38 (3)	C31—C32	1.36 (4)
C22—C27	1.47 (3)	C31—C33^i^	1.37 (3)
C23—C24	1.37 (4)	C31—H31	0.9500
C23—H23	0.9500	C32—C33	1.42 (3)
C24—C25	1.38 (3)	C32—H32	0.9500
C24—H24	0.9500	C33—H33	0.9500
C25—C26	1.34 (3)		
			
C1—B1—I1	117.7 (11)	C272—C27—C22	109.4 (17)
C1—B1—I2	112.0 (11)	C272—C27—C271	105.5 (19)
I1—B1—I2	106.6 (7)	C22—C27—C271	114 (2)
C1—B1—I3	105.6 (10)	C272—C27—H27	109.2
I1—B1—I3	107.0 (8)	C22—C27—H27	109.2
I2—B1—I3	107.3 (7)	C271—C27—H27	109.2
C2—N1—C1	110.1 (12)	C26—C28—C282	113.6 (16)
C2—N1—C11	118.4 (12)	C26—C28—C281	108.5 (16)
C1—N1—C11	131.3 (11)	C282—C28—C281	107.7 (16)
C1—N2—C3	110.5 (13)	C26—C28—H28	109.0
C1—N2—C21	130.4 (12)	C282—C28—H28	109.0
C3—N2—C21	118.3 (12)	C281—C28—H28	109.0
N2—C1—N1	105.7 (12)	C17—C171—H17A	109.5
N2—C1—B1	128.7 (14)	C17—C171—H17B	109.5
N1—C1—B1	125.1 (12)	H17A—C171—H17B	109.5
C3—C2—N1	106.8 (13)	C17—C171—H17C	109.5
C3—C2—H2	126.6	H17A—C171—H17C	109.5
N1—C2—H2	126.6	H17B—C171—H17C	109.5
C2—C3—N2	106.8 (12)	C17—C172—H17D	109.5
C2—C3—H3	126.6	C17—C172—H17E	109.5
N2—C3—H3	126.6	H17D—C172—H17E	109.5
C12—C11—C16	124.7 (15)	C17—C172—H17F	109.5
C12—C11—N1	117.1 (14)	H17D—C172—H17F	109.5
C16—C11—N1	117.4 (14)	H17E—C172—H17F	109.5
C11—C12—C13	116.5 (16)	C18—C181—H18A	109.5
C11—C12—C17	123.8 (15)	C18—C181—H18B	109.5
C13—C12—C17	119.7 (15)	H18A—C181—H18B	109.5
C12—C13—C14	121.6 (16)	C18—C181—H18C	109.5
C12—C13—H13	119.2	H18A—C181—H18C	109.5
C14—C13—H13	119.2	H18B—C181—H18C	109.5
C15—C14—C13	118.6 (18)	C18—C182—H18D	109.5
C15—C14—H14	120.7	C18—C182—H18E	109.5
C13—C14—H14	120.7	H18D—C182—H18E	109.5
C16—C15—C14	122 (2)	C18—C182—H18F	109.5
C16—C15—H15	118.9	H18D—C182—H18F	109.5
C14—C15—H15	118.9	H18E—C182—H18F	109.5
C15—C16—C11	116.3 (17)	C27—C271—H27A	109.5
C15—C16—C18	121.6 (16)	C27—C271—H27B	109.5
C11—C16—C18	122.1 (15)	H27A—C271—H27B	109.5
C172—C17—C12	112.2 (16)	C27—C271—H27C	109.5
C172—C17—C171	110.3 (15)	H27A—C271—H27C	109.5
C12—C17—C171	108.2 (17)	H27B—C271—H27C	109.5
C172—C17—H17	108.7	C27—C272—H27D	109.5
C12—C17—H17	108.7	C27—C272—H27E	109.5
C171—C17—H17	108.7	H27D—C272—H27E	109.5
C182—C18—C181	111.5 (16)	C27—C272—H27F	109.5
C182—C18—C16	112.7 (15)	H27D—C272—H27F	109.5
C181—C18—C16	108.9 (13)	H27E—C272—H27F	109.5
C182—C18—H18	107.9	C28—C281—H28A	109.5
C181—C18—H18	107.9	C28—C281—H28B	109.5
C16—C18—H18	107.9	H28A—C281—H28B	109.5
C22—C21—C26	124.4 (16)	C28—C281—H28C	109.5
C22—C21—N2	119.9 (16)	H28A—C281—H28C	109.5
C26—C21—N2	115.3 (15)	H28B—C281—H28C	109.5
C21—C22—C23	115 (2)	C28—C282—H28D	109.5
C21—C22—C27	122.8 (18)	C28—C282—H28E	109.5
C23—C22—C27	122 (2)	H28D—C282—H28E	109.5
C24—C23—C22	122 (2)	C28—C282—H28F	109.5
C24—C23—H23	118.9	H28D—C282—H28F	109.5
C22—C23—H23	118.9	H28E—C282—H28F	109.5
C23—C24—C25	120 (2)	C32—C31—C33^i^	120 (2)
C23—C24—H24	120.1	C32—C31—H31	119.8
C25—C24—H24	120.1	C33^i^—C31—H31	119.8
C26—C25—C24	123 (2)	C31—C32—C33	123 (2)
C26—C25—H25	118.7	C31—C32—H32	118.6
C24—C25—H25	118.7	C33—C32—H32	118.6
C25—C26—C21	115.7 (19)	C31^i^—C33—C32	117 (2)
C25—C26—C28	121.9 (18)	C31^i^—C33—H33	121.7
C21—C26—C28	122.4 (15)	C32—C33—H33	121.7
			
C3—N2—C1—N1	3.0 (17)	C11—C12—C17—C172	−122.6 (18)
C21—N2—C1—N1	−166.1 (16)	C13—C12—C17—C172	56 (2)
C3—N2—C1—B1	−169.6 (15)	C11—C12—C17—C171	115.4 (18)
C21—N2—C1—B1	21 (3)	C13—C12—C17—C171	−66 (2)
C2—N1—C1—N2	−2.5 (17)	C15—C16—C18—C182	−32 (2)
C11—N1—C1—N2	172.8 (14)	C11—C16—C18—C182	145.9 (16)
C2—N1—C1—B1	170.5 (14)	C15—C16—C18—C181	91.9 (19)
C11—N1—C1—B1	−14 (2)	C11—C16—C18—C181	−89.8 (19)
I1—B1—C1—N2	−162.7 (13)	C1—N2—C21—C22	−102 (2)
I2—B1—C1—N2	−39 (2)	C3—N2—C21—C22	89.3 (18)
I3—B1—C1—N2	77.9 (17)	C1—N2—C21—C26	85 (2)
I1—B1—C1—N1	26 (2)	C3—N2—C21—C26	−83.5 (18)
I2—B1—C1—N1	150.2 (12)	C26—C21—C22—C23	−9 (2)
I3—B1—C1—N1	−93.3 (15)	N2—C21—C22—C23	178.6 (15)
C1—N1—C2—C3	0.9 (17)	C26—C21—C22—C27	167.8 (15)
C11—N1—C2—C3	−175.0 (13)	N2—C21—C22—C27	−4 (2)
N1—C2—C3—N2	0.9 (17)	C21—C22—C23—C24	5 (3)
C1—N2—C3—C2	−2.5 (18)	C27—C22—C23—C24	−172.3 (19)
C21—N2—C3—C2	168.1 (14)	C22—C23—C24—C25	0 (3)
C2—N1—C11—C12	−79.4 (18)	C23—C24—C25—C26	0 (3)
C1—N1—C11—C12	105.6 (18)	C24—C25—C26—C21	−4 (3)
C2—N1—C11—C16	90.4 (16)	C24—C25—C26—C28	174.6 (19)
C1—N1—C11—C16	−84.6 (19)	C22—C21—C26—C25	9 (2)
C16—C11—C12—C13	3 (2)	N2—C21—C26—C25	−178.7 (14)
N1—C11—C12—C13	172.0 (13)	C22—C21—C26—C28	−169.4 (16)
C16—C11—C12—C17	−178.5 (15)	N2—C21—C26—C28	3 (2)
N1—C11—C12—C17	−9 (2)	C21—C22—C27—C272	−102 (2)
C11—C12—C13—C14	0 (2)	C23—C22—C27—C272	75 (3)
C17—C12—C13—C14	−178.8 (17)	C21—C22—C27—C271	140.1 (18)
C12—C13—C14—C15	−2 (3)	C23—C22—C27—C271	−43 (2)
C13—C14—C15—C16	1 (3)	C25—C26—C28—C282	46 (2)
C14—C15—C16—C11	2 (3)	C21—C26—C28—C282	−135.5 (16)
C14—C15—C16—C18	179.9 (17)	C25—C26—C28—C281	−73 (2)
C12—C11—C16—C15	−4 (2)	C21—C26—C28—C281	104.9 (17)
N1—C11—C16—C15	−172.7 (13)	C33^i^—C31—C32—C33	5 (5)
C12—C11—C16—C18	178.0 (14)	C31—C32—C33—C31^i^	−5 (5)
N1—C11—C16—C18	9 (2)		

Symmetry code: (i) −*x*+1, −*y*+1, −*z*+1.

### Hydrogen-bond geometry (Å, º)

**Table:**

*D*—H···*A*	*D*—H	H···*A*	*D*···*A*	*D*—H···*A*
C2—H2···I2^ii^	0.95	3.03	3.773 (13)	136

Symmetry code: (ii) *x*, −*y*+3/2, *z*+1/2.
